# L-Dopa and the Albino Riddle: Content of L-Dopa in the Developing Retina of Pigmented and Albino Mice

**DOI:** 10.1371/journal.pone.0057184

**Published:** 2013-03-19

**Authors:** Suzanne Roffler-Tarlov, Jin Hong Liu, Elena N. Naumova, Maria Margarita Bernal-Ayala, Carol A. Mason

**Affiliations:** 1 Department of Neuroscience, Tufts University School of Medicine, Boston, Massachusetts, United States of America; 2 Department of Anatomy and Cell Biology, Tufts University School of Medicine, Boston, Massachusetts, United States of America; 3 Department of Civil and Environmental Engineering, Tufts University, Medford, Massachusetts, United States of America; 4 Department of Pathology and Cell Biology, College of Physicians and Surgeons, Columbia University, New York, New York, United States of America; 5 Department of Neuroscience, College of Physicians and Surgeons, Columbia University, New York, New York, United States of America; 6 Department of Ophthalmology, College of Physicians and Surgeons, Columbia University, New York, New York, United States of America; The University of Melbourne, Australia

## Abstract

**Background:**

The absence or deficiency of melanin as in albinos, has detrimental effects on retinal development that include aberrant axonal projections from eye to brain and impaired vision. In pigmented retinal pigment epithelium (RPE), dihydroxyphenalanine (L-Dopa), an intermediate in the synthetic path for melanin, has been hypothesized to regulate the tempo of neurogenesis. The time course of expression of retinal L-Dopa, whether it is harbored exclusively in the RPE, the extent of deficiency in albinos compared to isogenic controls, and whether L-Dopa can be restored if exogenously delivered to the albino have been unknown.

**Methodology/Principal Findings:**

L-Dopa and catecholamines including dopamine extracted from retinas of pigmented (*C57BL/6J)* and congenic albino (*C57BL/6J-tyr^c2j^*) mice, were measured throughout development beginning at E10.5 and at maturity. L-Dopa, but not dopamine nor any other catecholamine, appears in pigmented retina as soon as tyrosinase is expressed in RPE at E10.5. In pigmented retina, L-Dopa content increases throughout pre- and postnatal development until the end of the first postnatal month after which it declines sharply. This time course reflects the onset and completion of retinal development. L-Dopa is absent from embryonic albino retina and is greatly reduced in postnatal albino retina compared to pigmented retina. Dopamine is undetectable in both albino and pigmented retinas until after the postnatal expression of the neuronal enzyme tyrosine hydroxylase. If provided to pregnant albino mothers, L-Dopa accumulates in the RPE of the fetuses.

**Conclusions:**

L-Dopa in pigmented RPE is most abundant during development after which content declines. This L-Dopa is not converted to dopamine. L-Dopa is absent or at low levels in albino retina and can be restored to the RPE by administration *in utero*. These findings further implicate L-Dopa as a factor in the RPE that could influence development, and demonstrate that administration of L-Dopa could be a means to rescue developmental abnormalities characteristic of albinos.

## Introduction

The mature neural retina (NR) is a layered sheet of photoreceptors, interneurons, projection neurons (retinal ganglion cells) and radial glial cells. At the back of the NR is a single layer of pigmented epithelial cells, the retinal pigment epithelium (RPE). During prenatal and postnatal development, retinal neurons divide adjacent to the RPE. The different retinal cells types are generated there in an orderly sequence [1]. In albinism, the biogenesis and/or packaging of the melanin pigment in the RPE is defective and, as a consequence, the development of the adjacent NR is flawed. Albinism is caused by a variety of genetic defects including mutations in the gene that codes for tyrosinase, the enzyme necessary for melanin synthesis [2]–[6]. Defects in pigment production are accompanied by faulty development of the NR: there are reports of an initial surplus of mitotic cells [7], a disrupted pace of neurogenesis [8], lack of cells in the fovea, and reduction in the numbers of uncrossed retinal ganglion cell axons to central targets [9]–[12]. The consequences of the albino developmental phenotype for human vision include loss of binocular vision, photophobia, nystagmus, and severe reduction of visual acuity [13].

The connection between failure of melanogenesis due to defects in tyrosinase and the development of the NR is supported by correction in the visual projections to the thalamus after replacement of the tyrosinase mini-gene in mice [14]–[16]. The pathology displayed by the albino's NR is linked to alterations in the pigment pathway in the RPE, but the relationship between the RPE, factors produced by the RPE, and NR development are poorly understood.

L-Dopa is a vitally important amino acid generated by the actions of two different enzymes on tyrosine. They are tyrosinase (TYR) in the pigment pathway and tyrosine hydroxylase (TH) in sympathetic neurons, in the adrenal glands, and in the brain's dopaminergic and noradrenergic neurons. In the pigment pathway, L-Dopa is generated at two different points in the cycle of melanin production [17].

In neurons and chromaffin cells, the catecholamine transmitters (dopamine, norepinephrine and epinephrine) accumulate but their precursor L-Dopa does not. This is due to the rapid conversion of L-Dopa to dopamine by aromatic amino acid decarboxylase (aadc): L-Dopa is usually undetectable unless aadc is blocked. Thus, within catecholamine-containing neurons, L-Dopa appears to be strictly an intermediate in the synthetic chain.

Our previous studies suggest that L-Dopa formed by TYR activity has a more complex role than that formed by TH and rapidly metabolized in neurons. We found that low levels of catecholamines were produced in pigmented neonatal TH-null mutant mice in spite of their lack of functional TH. We then made a double mutant that lacked both TH and TYR. No catechols were produced by the double mutants showing that the catecholamines produced in the absence of TH, resulted from the activity of TYR [18]. Subsequently, we uncovered a period of transient production of both L-Dopa and dopamine in peripheral tissues of mouse neonates that is the consequence of TYR activity, and not TH activity [19]. We also discovered that the TYR-derived catechols have biologic activity that can direct development [20]. Questions arising from these earlier studies include whether L-Dopa formed in the melanin pathway in the RPE is also subject to conversion to dopamine through aadc and whether L-Dopa, when it is TYR-derived, is maintained at high levels, suggesting a function in retinal development.

These questions prompted the present study which compares albino (TYR-null) and pigmented mouse retina to determine whether L-Dopa and/or dopamine are synthesized and accumulate in the retina, and if so where: in the RPE and/or the NR. We reasoned that if either or both of these catechols are present in developing pigmented RPE and are not present in the albino, they would be candidates for developmental signals for the adjacent NR as previously proposed, and for the development and function of the RPE itself [7], [21], [22]. Here we demonstrate that L-DOPA is found in the retinas of pigmented fetuses and neonates. By contrast, L-Dopa is rarely detectable in the albino retina prenatally. After birth, L-Dopa is present in the albino's retina but not at the levels seen in pigmented retina. Second, L-Dopa is found primarily in the RPE. Finally, we demonstrate that feeding L-DOPA to pregnant albino mice results in the appearance and accumulation of L-DOPA in fetal retinas.

## Results

### Whereas L-Dopa content increases throughout fetal development in pigmented retina, albino fetal retinas develop without L-Dopa

**Figure 1:**
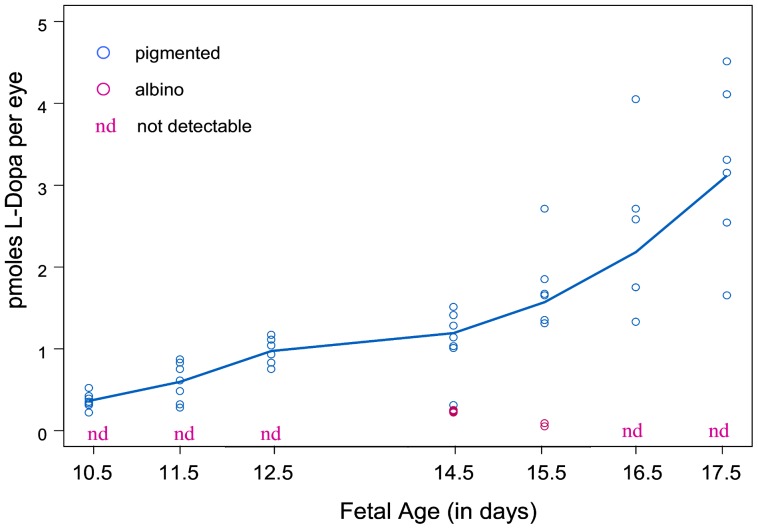
The Content of L-Dopa in Fetal Pigmented and Albino Eyes from Embryonic Day 10.5 to 17.5. The L-Dopa content of individual eye samples appears on the graph according to fetal age. Fetal eyes were extracted for amine content at 7 ages ranging from E10.5, the day of first tyrosinase expression, to E17.5. Samples contained as few as the two eyes of a single fetus to as many as 12. L-Dopa was measured in every pigmented eye sample from E10.5 onward. No additional catecholamines were found. The quantity of L-Dopa in individual samples extracted from pigmented eyes is represented by the blue circles on the graph. Little or no L-Dopa was found in the extracts of albino eyes at any age (marked as “nd”, not detectable). The pink circles are the values from the three albino eye samples in which traces of L-Dopa were detectable. The solid blue line, obtained by non-parametric smoothing, reflects the temporal trend in the L-Dopa values for pigmented eyes. Also evident is the increased variability in L-Dopa content as the pigmented fetuses grow.

To determine the time course of L-DOPA production during retinal development, we compared L-DOPA levels in pigmented and albino eyes. Figure 1 shows L-Dopa concentrations (pmoles per eye) in whole eyes without the lens from pigmented and albino fetuses taken at intervals starting at E10.5 until shortly before birth. L-Dopa was first detectable in fetal pigmented eyes at E10.5 (fig. 1) the day that tyrosinase is first expressed in mice and one day before pigment is visible in the developing RPE [23]. L-Dopa levels increased thereafter throughout prenatal development. L-Dopa was the only catechol detectable in pigmented fetal eyes. Dopamine was undetectable in both embryonic and early postnatal pigmented retinas.

In contrast to fetal pigmented eyes, the fetal albino eyes displayed little or no production and accumulation of L-Dopa during embryonic development. Although equivalent numbers of pigmented and albino eye samples were tested at every age, traces of L-Dopa were detectable in only three samples of eyes from fetal albinos (fig. 1). We analyzed samples that contained up to 12 albino eyes late in gestation and found no L-Dopa, whereas L-Dopa was detectable in eyes of every individual pigmented fetus. Dopamine was not found in any of the fetal eyes, pigmented or albino.

**Figure 2:**
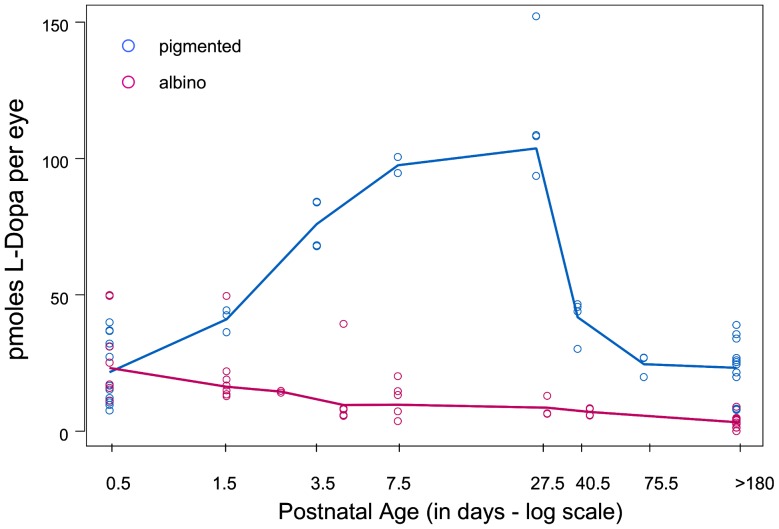
The Content of L-Dopa in Pigmented and Albino Retinas after Birth. To better illustrate the very rapid initial changes in L-Dopa concentrations in pigmented retinas that occur postnatally, the L-Dopa values are plotted on a log-transformed scale for postnatal age. L-Dopa content of individual samples of pigmented retina are represented by blue circles. The pink circles are the L-Dopa values obtained for individual samples of albino retinas. The solid lines obtained by non-parametric smoothing reflect temporal trends in the L-Dopa values for pigmented eyes (blue line) and for albino retinas (pink line). The content of L-Dopa in pigmented retinas rises throughout the period of postnatal retinal development and reaches maximum values by 27.5 days and then declines. Values were equivalent for albino and pigmented eyes on the day of birth (P0.5). After that, L-Dopa was detectable in most but not all albino retinas but content declined throughout postnatal retinal development rather than increased with postnatal age as occurred in the pigmented retina.

The comparison of endogenous L-Dopa content between albino and pigmented retinas during postnatal development is shown in Figure 2. Also evident in the chart is the variability of L-Dopa levels in retinas of pigmented pups of the same age. L-Dopa content of both pigmented and albino retinas rose abruptly at birth when content of L-Dopa was equivalent in the albino and pigmented retinas. As development proceeded during the first postnatal days, L-DOPA levels rapidly increased in the pigmented retinas, whereas the L-Dopa content of the albino retinas declined (fig. 2).

After the first postnatal month when development and differentiation of retina were complete, the concentration of L-Dopa in the pigmented retina dropped but never disappeared (fig. 2). The L-Dopa content in adult pigmented retina (180 days and older) was the same as at birth but remained higher than that found in the albino retinas at the same age.

### The compartmentalization of L-Dopa in the retina: L-Dopa in pigmented retina of fetal and mature eyes is sequestered largely in the RPE

To determine whether L-DOPA is located in the RPE and/or the NR, the two retinal components were separated and analyzed individually for catechol content. This experiment was carried out in pigmented and albino mice at two ages: E14.5, four days after tyrosinase is expressed in RPE and at the end of the first postnatal month when proliferation and differentiation of the NR is complete. Pigmented eyes were taken from 10 fetuses from three different litters. The albino eyes were also taken from 10 fetuses that came from three different litters. The two parts of mature retina were separated in eyes of 16 pigmented mice (aged P27.5 to P 31.5) and 13 albino mice (aged P30.5). In all cases, the two NR from a single animal were combined for extraction as were the two RPE. Table 1 reports the numbers of RPE and NR samples that were positive for L-Dopa at both ages of pigmented and albino and the concentrations of L-Dopa (as pmoles per single RPE or NR) where it was present.

**Table 1:** Compartmental location of L-Dopa in retina: in retinal pigment epithelium (RPE) and/or neural retina (NR): prenatal and postnatal, pigmented and albino.

		Pigmented		Albino	
Retinal Compartment	Age	L-Dopa positive of total	mean ± SEM	L-Dopa positive of total	mean ± SEM
**RPE**	Prenatal^1^	10/10	1.6+/−0.2	0/10	
	Postnatal^2^	16/16	132+/−6	4/13	1.45+/−0.6
**NR**	Prenatal^1^	0/10		0/10	
	Postnatal^2^	2/16	14	0/13	

1 E14.5, ^2^P27.5–P31.5

The retina's L-Dopa was found in every pigmented fetal RPE tested, whereas none was detected in the NR of the same retinas. Neither the NR nor the RPE taken from fetal albinos was positive for L-Dopa. Similarly, L-Dopa was present in all of the postnatal pigmented retinas where it was heavily concentrated in RPE. Every pigmented RPE contained L-Dopa. L-Dopa was found in only two of the 16 pigmented NR. The small amount of L-Dopa detected in postnatal albino retinas was in the RPE: none was in NR. Thus, L-Dopa made in the pigment pathway in retina is sequestered in the RPE.

### L-Dopa made in the RPE of pigmented retina is not converted to dopamine

#### Dopamine is undetectable in developing retina of both pigmented and albino

Whether L-Dopa in the developing pigmented retina is converted to dopamine is a key question as dopamine seems a more likely signaling candidate than does L-Dopa. Dopamine receptors are present in NR [24] and dopamine receptors are not activated by L-Dopa (Roffler-Tarlov, unpublished). Thus, we analyzed retinal and eye samples for dopamine. No dopamine was detected in either pigmented or albino fetal eyes nor in postnatal retina up to three weeks of age.

### Dopamine appears in retina 3–4 weeks after birth and is equivalent in pigmented and albino retina

Three to four week-old pigmented and albino retinas contained dopamine. The timing is coincident with that of TH expression and maturation of amacrine cells [25]. Both albino and pigmented mice express TH and the concentrations of dopamine were equivalent in pigmented and albino retinas of month-old mice. Dopamine content was 6.66+/−0.50 pmoles per pigmented retina (n = 13 retinas) and 6.00+/−0.50 pmoles per albino retina (n = 10 retinas) (means +/− SEM). These results support the view that all dopamine in the retina is a consequence of the activity of TH in the neuronal pathway in NR and that L-Dopa made in RPE for the melanin pathway is not converted to dopamine.

### L-Dopa administered to pregnant dams accumulates in the eyes of their fetuses

We next investigated whether L-Dopa can be restored to albino fetal retinas and whether the L-Dopa content of the pigmented fetal retinas can be altered. L-Dopa content was assessed in the retinas (RPE and NR included) of three litters of albino fetuses and pigmented fetuses whose mothers drank water that contained L-Dopa beginning on E10.5. The eyes of the L-Dopa treated fetuses were taken at E14.5. Eyes were also examined at E14.5 from untreated albino and untreated pigmented fetuses for comparison.

No L-Dopa was detected in eyes from two of the three litters of untreated albino fetuses examined as shown in Table 2. Traces of L-Dopa were found in eyes of 3 of 6 fetuses from one of the untreated albino litters. In contrast, every albino fetal eye from the three L-Dopa-treated albino litters contained L-Dopa. The quantity of L-DOPA in the treated albino eyes was roughly equivalent to that in untreated pigmented eyes of the same age (Table 2). L-Dopa treatment of the pregnant pigmented mother increased the normal levels of L-Dopa in pigmented eyes to a variable extent as shown.

**Table 2:** Accumulation of exogenous L-Dopa by albino and pigmented fetal retinas: Comparison.

Retinal phenotype	Treatment	Number of retinas positive for L-Dopa	pmoles L-Dopa per retina
			*mean* ± *SEM*
**Albino**	Untreated – litter 1	3/6	0.4±0.2
	Untreated – litter 2	0/3	---------
	Untreated – litter 3	0/5	---------
**Albino**	L- Dopa treated – litter 1	6/6	1.5±0.2
	L-Dopa treated – litter 2	8/8	3.0±0.3
	L-Dopa treated – litter 3	5/5	2.8±0.6
**Pigmented**	Untreated – litter 1	6/6	2.0±0.5
	Untreated – litter 2	3/3	2.2±0.2
	Untreated – litter 3	5/5	1.8±0.4
**Pigmented**	L-Dopa treated – litter 1	3/3	6.0±2.0
	L-Dopa treated – litter 2	5/5	16.8±9.1

Whereas dopamine was undetectable in untreated eyes from both pigmented and albino fetuses, dopamine was found in half of the L-Dopa-treated fetal eyes; pigmented and albino. The amount of dopamine made varied greatly among the eyes examined; values ranged from 0.3 to 12.9 pmoles/eye in both pigmented and albino. Thus, exogenously supplied L-Dopa can reach sites in neural retina where it is converted to dopamine by the aromatic amino acid decarboxylase present there [26].

## Discussion

### The time course of L-Dopa accumulation in the RPE during the development of pigmented and albino retinas

Our data show that L-Dopa is both synthesized and accumulated in the RPE of pigmented mouse retinas, appearing at E10.5 concomitant with the first expression of the tyrosinase gene in RPE [23]. L-Dopa is not detectable in NR nor is it converted to dopamine. L-Dopa content rises steadily throughout prenatal development while ganglion cells complete mitosis and amacrine cells and photoreceptors are generated. After birth, L-Dopa content increases further throughout the first four postnatal weeks during the generation of amacrine, horizontal, bipolar, Mueller cells, and rod photoreceptors. The rise of L-Dopa overlaps the entire period of genesis and differentiation of cells in the NR and then L-Dopa content drops but does not disappear. These data are compatible with earlier hypotheses that L-Dopa has a part in a signaling cascade essential for the normal generation of neural cells. In the albino, during this period when L-Dopa is absent or at very low levels, neural cell generation is perturbed [7], [8], [27].

Fetal albino retinas develop without L-Dopa. This result emphasizes that all L-Dopa in pigmented retina during embryonic and postnatal ages is formed exclusively by TYR activity, absent in the albinos. On the day of birth, the quantity of L-Dopa extracted from albino retina rises sharply from near absence to levels equivalent to those in pigmented retina. The sudden appearance of L-Dopa in the albino's retina as well as its rapid rise in pigmented retinas are likely the consequence of the catecholamine surge fueled by activity of the neuronal enzyme TH in the adrenal gland that occurs at birth in both albino and pigmented animals [26]. Following birth, L-Dopa content diverges dramatically between pigmented and albino retinas, declining in albino and rising in pigmented retina during the generation and differentiation of rod photoreceptors, horizontal cells, and Mueller cells.

### The Influence of L-Dopa and dopamine on neural proliferation

Both L-Dopa and dopamine have been proposed as mitotic regulators based on patterns of mitosis in the retinas of albino and pigmented rats [7] and on results of *in vitro* studies of L-Dopa and dopamine applied exogenously to cultured albino and pigmented retinas [21], [27], [31]. Ilia and Jeffrey found elevated numbers of thymidine-labeled cells pointing to increased mitotic activity in NR in mature albino rats compared to pigmented [7], [27]. Abnormal mitotic spindle orientation of cells in the albino was corrected with injection of L-Dopa [28]. Tyrosine hydroxylase has been ectopically expressed in albino RPE *in vivo*, with the intent of providing L-Dopa to that site without producing melanin. The authors reported rescue of the albino's ganglion cell projection abnormality, although production of L-Dopa was not directly demonstrated [29]. It has also been reported that a single subcutaneous injection of L-Dopa into an albino rat pup corrects the elevated rate of mitosis in NR and the increased expression of gap junction protein Cx-43, the latter of potential importance in communication with the NR and RPE [30].

In contrast to L-Dopa, we found no dopamine in pigmented retina until several weeks after birth. Dopamine appears after expression of the neuronal enzyme TH by dopamine-containing amacrine cells [24]. Both albino and pigmented lines express TH and there was equivalent dopamine content in albino and pigmented retina. Thus L-Dopa formed much earlier in the pigment pathway in RPE through the activity of tyrosinase is not subsequently converted to dopamine. This is important because both L-Dopa and dopamine have been implicated in regulating mitotic activity of retinal (see below). Our finding that dopamine is absent during the period of mitotic activity in the pigmented retina indicates that it cannot have such a role.

*In vitro* experiments in which L-Dopa was provided to isolated chick retinas do show robust conversion of L-Dopa to dopamine [21]. The content of dopamine increased 100 fold after exposure of retinal segments (cultured without RPE) from E9 chick embryos to exogenous L-Dopa in the culture medium. When applied to NR cultured from rat pups, dopamine prolonged mitosis in the albino's NR but not in NR from the pigmented retina [22]. However, the experiments that show mitotic activity elicited by dopamine *in vitro* do not reflect *in vivo* conditions. The disagreement between the outcomes of *in vitro* studies and the results of the *in vivo* studies reported here is likely due to the difference in the proximity of L-Dopa to aadc, the enzyme that decarboxylates L-Dopa to form dopamine. When exogenous L-Dopa is provided to cultured retina, the L-Dopa readily reaches the sites of aadc activity in NR. L-Dopa sequestered in RPE *in vivo* is not available for decarboxylation because aadc is present only in NR [25]. Postnatally, dopamine content of albino and pigmented retina is equivalent due to activity of neuronal TH present in retinas whether or not pigment is made. The appearance of dopamine late postnatally most likely marks the maturation and functioning of dopamine-containing amacrine cell vesicles in NR that takes place after the eyes open [24], [32], [33].

### Delivery of L-Dopa to the albino's retina *in utero*

If L-Dopa participates in a signaling pathway that is a key to normal development, it could be helpful to supply exogenous L-Dopa to retinas in which it is lacking. We found that it is possible to manipulate L-Dopa levels in the fetal eye. When the drinking water of the pregnant albino dam was laced with L-Dopa, L-Dopa appeared in the eyes of her fetuses. Albino fetuses treated in this way throughout prenatal development accumulate L-Dopa content equivalent to that of the untreated pigmented eye measured at E14.5 (see Table 2). Pigmented fetal eyes were also affected by L-Dopa fed to the mothers. These accumulated greater-than-normal quantities of L-Dopa that were extremely variable (Table 2). About half of the L-Dopa-treated pigmented eyes as well as albino eyes did form dopamine which demonstrates that exogenous L-Dopa supplied *in utero* can reach the NR where becomes a substrate for aadc.

The sole example to date of the potential usefulness of L-Dopa treatment *in utero* for the eye was provided by investigations of modifier genes that contribute to the effects of congenital glaucoma in mouse models of the human disease [34]. A structural abnormality of ocular drainage was made worse on a tyrosinase-deficient background, the *C57BL/6J-tyr^c2j^/tyr^c2^* mouse used here. The deficits compounded by tyrosinase deficiency were not seen after L-Dopa treatment *in utero,* as performed here.

### Lack of L-Dopa in the albino retina and consequences for cell fate and differentiation

That the decline of L-Dopa in pigmented retina occurs after the generation and differentiation of retinal neurons suggests that it serves a special function during the fetal and early postnatal period. One hypothesis is that L-Dopa entrains cell division, and the timing of cell division would regulate the transcription factors directing retinal cell fates (retinal ganglion cells vs amacrine cells, for example). In the albino, fewer cells express Zic2, the transcription factor associated with the projection of retinal ganglion cell axons ipsilaterally [35]–[37]. Studies are in progress to determine whether L-Dopa provided to fetuses can correct misspecification of retinal ganglion cells.

The present data facilitate answering the essential issue in albinism: whether defects in vision characteristic of albino mammals can be traced to abnormal communication during eye development between the retinal layers: the retinal pigment epithelium (RPE) and the neural retina (NR) and involving factors such as L-Dopa. How would that take place? An intriguing finding is that L-Dopa may be the ligand for the orphan G-protein-coupled receptor on melanosomes that is mutated in OA1 albinism [38]. If this is the case, L-Dopa may be essential for the operation of a signaling pathway in RPE and in turn, the production of factors that affect retinal development.

## Methods

### Ethics Statement

All experiments were conducted with an approved protocol from the Tufts University Institutional Animal Care and Use Committee.

### Mice

We examined mice from two lines of the C57BL/6J strain, one albino and one pigmented. These albino and pigmented mice are genetically identical except for a spontaneously occurring point mutation of the Tyr gene. Mice with two copies of mutated Tyr (*C57BL/6J-tyr^c2j^/tyr^c2^*) are albino. The mutation results in loss of enzymatic activity of tyrosinase in melanosomes [6]. We compared separate litters of fetuses and postnatal mice from albino and pigmented parents timed consistently, with E0.5 being the day of conception and P0.5 the day of birth. Mice were housed in a cycle of 14 hours of light (5 am to 7 pm) and 10 hours of darkness (7 pm to 5 am). The comparison of the congenic strains is important because the albino phenotype is affected by modifying genes [29].

Mating pairs of both mouse lines were originally purchased from the Jackson Laboratory, Bar Harbor ME.

### Treatment of fetal mice with L-Dopa

Pregnant females, albino and pigmented, were fed 1 mg/ml L-Dopa (Sigma) dissolved in the drinking water with ascorbate (2.5 mg/ml to prevent oxidation) from E0.5 until the fetuses were taken for examination.

### Dissection and separation of neural retina and RPE

At midmorning during the normal light cycle, fetuses were removed from anesthetized females by hysterotomy. Eyes of postnatal pups and adults were removed from anesthetized mice. The eyes were separated from the orbit with curved round-ended scissors and excess tissue removed under a dissecting microscope. In most experiments in which fetal eyes where examined, L-Dopa was extracted from the whole eye excluding the lens if it was mature enough to be removed. In most studies of postnatal eyes, the amines were extracted from retina (NR and RPE) except where noted. The retina was separated from the rest of the eye while submerged in PBS. An incision was made through the limbus and extended circumferentially to separate the cornea after which the iris, lens, and vitreous were removed leaving the retina for acid-extraction of amines.

To harvest the NR without RPE, eyes were transferred to a petri dish containing Dulbecco's Phosphate Buffered Saline without calcium and magnesium (Sigma) at room temperature. An incision was made through the limbus with a needle. This incision was extended circumferentially to separate the cornea with the use of curved micro-scissors. Subsequently, the iris, lens and vitreous were removed. The retinas stood at room temperature in the solution for 5 min (fetal retina) or 15 minutes (postnatal retina). The RPE was peeled away from NR allowing each to be analyzed separately.

### Extraction and quantitation of L-Dopa and Dopamine

The catechols were extracted after sonication of the eye tissue in 100–500 microliters cold 0.2N perchloric acid. The sonicated tissues in acid remained on ice for at least 1 hour to precipitate proteins after which the samples were centrifuged in the cold. The supernatant was removed and filtered using a 0.22 um filter tube (Millipore Corp.). We maintained very small volumes to avoid dilution of the amines, and all amines that appear in the sonicate are oxidized. Amines are separated and quantitated using High Performance Liquid Chromatography (HPLC) followed by electrochemical detection according to procedures described earlier e.g. [18]. The HPLC system uses an ESA reverse phase C-18 column coupled to an ESA Coulochem II electro-chemical detector and ESA Test mobile phase. With this procedure we can detect L-Dopa in eyes from a single pigmented fetus.
